# The National Dispensatory

**Published:** 1884-10

**Authors:** 


**The National Dispensatory.** Containing the Natural History, Chemistry, Pharmacy, Actions and Uses of Medicines, including those recognized in the Pharmacopœias of the United States, Great Britain and Germany, with numerous references to the French Codex. By Alfred Stille, M. D., LL. D., Professor Emeritus of the Theory and Practice of Medicine and of Clinical Medicine in the University of Pennsylvania, and John M. Maisch, Phar. D., Professor of Materia Medica and Botany in the Philadelphia College of Pharmacy, Secretary to the American Pharmaceutical Association. Third edition, thoroughly revised and greatly enlarged, with 311 illustrations. Price, in cloth, $7.25; leather, raised bands, $8; very handsome half Russia, raised bands and open back, $9. Philadelphia: Henry C. Lea's Son & Co.

When the National Dispensatory first appeared, in 1879, it was hailed as supplying a want that had long been felt in both the medical and pharmaceutical professions. Its accuracy, its fullness, its conciseness, the happy manner in which, while omitting all that was obsolete or merely curious, it gave all the information that the practitioner or druggist could desire, not only with regard to the selection, preparation and compounding of drugs, but their physiological effects, their therapeutical use and their clinical value, gave it at once an unappoached position as a standard work and an indispensable book of reference. The exhaustion, in less than six months, of an unusually large edition was the most emphatic testimony of its practical value. The carefully revised second edition fully maintained its reputation, and so thoroughly was it brought up to the day that when, in 1882, the new United States Pharmacopœia appeared, it contained not more than half a dozen articles which had not been carefully treated by the authors of the Dispensatory.

Since then the authors have labored incessantly at the work with the view of making the third edition an even more complete representative of the science of 1884 than its first edition was of that of 1879. For this, ample material has been afforded not only by the new United States Pharmacopœia, but by those of Germany and France, which have recently appeared and have been incorporated in it, besides a large number of new non-officinal remedies. It is thus rendered the representative of the most advanced state of American, English, French and German pharmacology and therapeutics. The vast amount of new and important material thus introduced may be gathered from the fact that the additions to this edition amount, in themselves, to the matter of an ordinary full-sized octavo volume, rendering the work larger by twenty-five per cent. than the last edition. The Therapeutic Index, so suggestive and convenient to the practitioner, contains 1,600 more references than in the last edition—the general index over 3,700 more, rendering the entire number in the volume 22,390, while the list of illustrations has been increased by 80, and a number of wood-cuts have been substituted for others that were not deemed satisfactory.

Yet these facts inadequately represent the amount of labor bestowed on the revision, for it has not simply consisted in making additions. Many of the old articles have been condensed, others have been virtually re-written.

The work is wonderful in its completeness, and will maintain the position universally accorded to it as the standard authority in all matters pertaining to its subject, as registering the furthest advance of the science of the day, and as embodying, in a shape for convenient reference, the recorded results of experience in the laboratory, in the dispensing room, and at the bed-side.

